# PARADOCKS – a framework for molecular docking

**DOI:** 10.1186/1758-2946-3-S1-P35

**Published:** 2011-04-19

**Authors:** M Pippel, R Meier, W Sippl

**Affiliations:** 1 Institute of Pharmacy, Martin-Luther-University of Halle-Wittenberg, 06120 Halle (Saale), Germany; 2 Institute of Biochemistry, University of Leipzig, 04103 Leipzig, Germany

## 

The prediction of possible binding geometries as well as ranking of putative protein-ligand complexes according their binding affinities is the intention of so called molecular docking approaches. To evaluate complexes against each other, scoring functions are required. In recent years knowledge-based scoring functions have been evolved. They exploit the vast amount of experimentally determined structures to derive statistical atom pair potentials. In this work, the focus has been set on the implementation and the validation of the fast and robust knowledge-based objective function PMF04 into the molecular docking program PARADDOCKS (Parallel Docking Suite) [1]. PARADDOCKS is a flexible, easily extensible and open source docking program developed in our group.

For the implementation of the PMF04 scoring function we extracted the atom pair potentials from the publicly available potential of mean force (PMF) [2]. To avoid unfavourable docking conformations additional an vdW-term was added. To make the program more easy for developers to incorporate their own or adapted objective functions a Subgraph Search Description (PSSD) is implemented. It is a line notation to describe subgraph patterns similar to SMARTS. The primary difference is the lack of implicit hydrogen treatment and implicit bonds.

In the following, the performance of PARADDOCKS for virtual screening was compared with the commercially available docking program GOLD. Therefore 13 targets were selected from the directory of useful decoys (DUD) which contains a set of unbiased actives and decoys. Especially in terms of early enrichment PARADDOCKS outperforms the three available scoring functions implemented in GOLD.
